# Understanding Molecular Mechanisms of Durable and Non-durable Resistance to Stripe Rust in Wheat Using a Transcriptomics Approach

**DOI:** 10.2174/1389202911314020004

**Published:** 2013-04

**Authors:** Xianming Chen, Tristan Coram, Xueling Huang, Meinan Wang, Andrea Dolezal

**Affiliations:** 1 US Department of Agriculture, Agricultural Research Service, Wheat Genetics, Quality, Physiology and Disease Research Unit, Pullman, WA 99164-6430, USA; 2 Department of Plant Pathology, Washington State University, Pullman, WA 99164-6430, USA; 3 Dow AgroSciences LLC, 9330 Zionsville Road, Indianapolis, IN 46268, USA; 4 College of Plant Protection, Northwest A&F University, Yangling, Shaanxi, 712100, China

**Keywords:** Durable resistance, Genechips, Gene expression, Microarray, *Puccinia striiformis*, Yellow rust.

## Abstract

Stripe rust of wheat, caused by *Puccinia striiformis* f. sp. *tritici*, continues to cause severe damage worldwide. Durable resistance is necessary for sustainable control of the disease. High-temperature adult-plant (HTAP) resistance, which expresses when the weather becomes warm and plants grow older, has been demonstrated to be durable. We conducted numerous studies to understand the molecular mechanisms of different types of stripe rust resistance using a transcriptomics approach. Through comparing gene expression patterns with race-specific, all-stage resistance controlled by various genes, we found that a greater diversity of genes is involved in HTAP resistance than in all-stage resistance. The genes involved in HTAP resistance are induced more slowly and their expression induction is less dramatic than genes involved in all-stage resistance. The high diversity of genes and less dramatic induction may explain durability and the incomplete expression level of HTAP resistance. Identification of transcripts may be helpful in identifying resistance controlled by different genes and in selecting better combinations of genes to combine for achieving adequate and durable resistance.

## 1 INTRODUCTION

Stripe rust (or yellow rust), caused by *Puccinia striiformis* Westend. f. sp. *tritici* Erikss. (*Pst*), is one of the most important diseases of wheat worldwide [1, 2]. The disease continues to cause severe damage in many wheat-producing regions. Control is preferably through resistant cultivars. However, cultivars that are developed for resistance to stripe rust often become susceptible as virulent races continue to evolve in the pathogen populations and spread from one region to another. Race-specific resistance, which usually provides complete protection throughout the entire growth cycle and therefore referred as all-stage resistance, is usually not durable when conferred by a single gene. In contrast, high-temperature adult-plant (HTAP) resistance, which expresses when the weather becomes warm and/or plants grow older, has proven to be non race-specific and durable [1, 3, 4]. Plants with only HTAP resistance are susceptible to all races at the seedling stage, but become resistant or less susceptible in later growth stages when temperatures increase. Typical HTAP resistance is identified through a four-way (seedling-low temperature, seedling-high temperature, adult plant-low temperature and adult plant-high temperature) test [1, 5]. We routinely screen wheat germplasm and breeding lines for HTAP resistance by testing seedlings with various *Pst* races at a low temperature profile (diurnal temperature cycle changing from 4°C at 2:00 a.m. to 20°C at 2:00 p.m.) and then test adult-plants with selected races that are virulent in the seedling tests under a high temperature profile (diurnal temperature cycle changing from 10°C at 2:00 a.m. to 30°C at 2:00 p.m.). HTAP resistance has been successfully used in the US Pacific Northwest to reduce severe damage from stripe rust since Dr. Orville Vogel released wheat cultivars Gaines and Nugaines in the early 1960s. However, HTAP resistance is not complete and the disease level is influenced by growth stage, temperature, and inoculum load [1]. Therefore, the best approach is to combine genes for effective all-stage resistance with those for HTAP resistance. Numerous genes conferring both types of resistance have been identified. Comparison of the gene structures and predicted proteins of cloned stripe rust resistance genes *Yr18*/*Lr34* and *Yr36* reported by Krattinger *et al.* [6] and Fu *et al.* [7], respectively, and unpublished *Yr10* (Laroche, personal communication) and candidate *Yr5* (Chen and associates, unpublished data), together with many race-specific resistance genes across many host species, leads to a hypothesis that resistances controlled by NBS-LRR genes may not be durable, and resistances controlled by non-NBS-LRR genes are more likely to be durable. These cloned genes are not the only genes that contribute to resistance; it is likely that each *Yr* gene regulates various defense and other functional genes to confer the final response phenotypes. Here, we compare our data in a series of transcriptomics studies previously published [8-13] and unpublished to understand molecular mechanisms of race-specific all-stage and nonrace-specific HTAP resistance with an ultimate goal of finding a molecular basis for durable resistance. 

## 2 COMPARISON OF TRANSCRIPTS INDUCED DURING RACE-SPECIFIC ALL-STAGE RESISTANCE MEDIATED BY *YR5* AND RACE NON-SPECIFIC HTAP RESISTANCE MEDIATED BY *YR39*

**Fig. (1):**
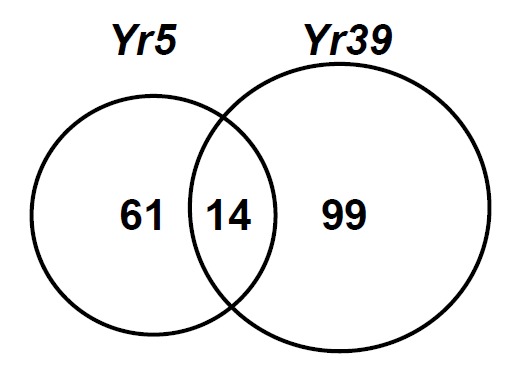
Comparison of numbers of transcripts associated with *Yr5*- mediated race-specific all-stage resistance (61) and *Yr39*-mediated race non-specific high-temperature adult-plant resistance (99). Fourteen of the genes were common to both types of resistance.

*Yr5* confers typical all-stage resistance to stripe rust, exhibiting infection type (IT) 0 (no visible symptoms) on leaves of the original donor, *Triticum spelta* album [14] and IT 1 (small necrotic flecks without uredinia) on the near-isogenic line (AvSYr5NIL) in the Avocet Susceptible (AvS) background [15]. In a study to determine genes involved in *Yr5*-controlled resistance [8, 9], the Wheat GeneChip (Affymetrix, Santa Clara, CA), which includes 61,127 probe sets representing 55,052 transcripts (www.affymetrix.com), was used to profile changes occurring in wheat near-isogenic lines (a BC_7_:F_4_ line of AvS x *Ts*a and AvS) at 6, 12, 24 and 48 h post-inoculation (hpi) with race PST-78 that is avirulent on the *Yr5* isoline and virulent on AvS. The microarray study identified 61 transcripts specific to *Yr5*-mediated resistance (Fig. **1**). These transcripts are genes typically involved in signaling pathways and defense-related events known to occur during R-gene-mediated responses, including protein kinase signaling and production of reactive oxygen species, leading to hypersensitive responses. The gene expression pattern showed a peak at 24 hpi, which is correlated to haustorial formation. In this study, 19 transcripts were identified to be specifically induced for basal defense during the compatible interaction. However, due to lack of *R*-gene signaling, the response was weak. In contrast, *Yr5*-signalling resulted in a rapid and strong resistance response.

In the study of *Yr39*-mediated HTAP resistance [10], the same Wheat GeneChip was used to identify genes induced in two selected F_7_ recombinant inbred lines (RILs) from a cross between AvS and Alpowa inoculated with PST-78 urediniospores and mock-inoculated without urediniospores at the flag-leaf stage and grown at the high temperature profile (10-30°C) after inoculation. The resistant RIL and Alpowa showed a typical HTAP resistance, IT 2-3 (necrotic stripes of 0.5-2.0 cm with occasional uredinia on the edges of necrotic stripes). Under the same temperature conditions, the susceptible RIL and AvS had IT 9 (uredinial stripes without chlorosis or necrosis). In this study, 99 induced transcripts were identified as HTAP resistance-specific (Fig. **1**). This number is higher than that specifically involved in *Yr5* resistance as discussed above. Transcript accumulation peaked at 48 hpi, which is later than the peak time (24 hpi) for *Yr5*-mediated all-stage resistance, but corresponded to the time point when rust hyphae were observed microscopically and were undergoing rapid increases in fungal biomass as detected by quantitative PCR assays in the compatible interaction. More than half (50.5%) of the annotated HTAP resistance transcripts were involved in defense and/or signal transduction, including *R*-gene homologs and transcripts associated with pathogenesis-related protein production, phenylpropanoid biosynthesis and protein kinase signaling. The identification of nine *R*-gene homologs leads to a hypothesis that these genes regulated by a master gene (*Yr39*) serve as secondary master genes regulating defense and other related genes contributing to HTAP resistance.

When we compared the *Yr39*-mediated HTAP resistance with the *Yr5*-mediated all-stage resistance, we found 14 genes involved in both types of resistance (Fig. **1**) [10]. These genes include WIR1A protein (involved in cell wall structure), beta-1,3-glucanase (a PR protein), phenylalanine ammonium lyase (a phenylpropanoid phytoalexin), peroxidase (involved in oxidative stress), protein kinase and calmodulin protein (involved in signal transduction), carbohydrate (related to transport), and blue copper-binding protein (related to electron transport) [10]. The common genes may be related to host cell death involved in both types of resistance. The putative functions of genes identified in *Yr39*-mediated resistance, but not in the *Yr5*-mediated resistance, included *R* proteins, UDP-glucosyl transferase and hydroxyanthranilate hydroxyl cinnamoyl transferase involved in phenylpropanoids, pleiotropic drug resistance/ABC transporter, putative disease resistance protein, latex protein allergen, receptor protein kinase involved in signal transduction, WRKY5 homolog involved in transcription, and amino acid/protein and ammonium/phosphate/ potassium involved in transportation. Some of the specific genes may explain the durability of the *Yr39*-mediated resistance, especially the R proteins. Three of the R protein genes are protein kinases with homology to RPG1 protein conferring durable resistance to stem rust in barley [16]. One *R* gene is a homolog of Cf2/Cf5 LRR disease resistance protein for *Cladosporium fulvum* resistance in tomato [17]. One putative *R* gene has homology with the putative stripe rust resistance protein *Yr10* (http://pir.uniprot.org/uniprot/Q9FR63). The remaining three *R* genes encode putative leucine-rich repeat family protein, leucine-rich repeat transmembrane protein kinase, and NB-ARC domain containing protein. In addition, a homolog of Hm1 NADPH-dependent HC-toxin reductase protein was involved in the *Yr39*-mediated resistance. *Hm1* conferring resistance to *Cochliobolus carbonum* in maize was the first cloned plant disease resistance gene [18]. The collective contribution of these *R* genes to the *Yr39*-mediated resistance may require different *Pst* genes for recognition. The diverse *R* genes may regulate various defense genes involved in different abiotic stress response pathways. All of the *R* and defense genes make the *Yr39*-mediated HTAP resistance diversely based, perhaps making it difficult for the pathogen to overcome. 

## 3 COMPARISON OF TRANSCRIPTS IDENTIFIED FOR DIFFERENT GENES CONFERRING RACE-SPECIFIC ALL-STAGE RESISTANCE

In a study aimed at identifying common transcripts associated with race-specific all-stage resistance [13], genes *Yr1*,* Yr5*,* Yr7*,* Yr8*,* Yr9*,* Yr10*,* Yr15 *and *Yr17* were selected because they are available in near-isogenic lines in AvS background. Due to budget limitations, we used a custom microarray instead of the Wheat Affymetrix GeneChip with a primary goal of identifying common transcripts. A total of 343 probes were selected based on their significant expression in the *Yr5* and *Yr39* studies [8, 10]. An avirulent race was used to inoculate two-leaf seedlings of each of the *Yr* gene lines and PST-78 was used to inoculate the susceptible background line AvS, with the same race being used for each gene when possible. A mock-inoculation was also used for each of the lines. The inoculated seedlings were grown at the low-temperature profile described in the *Yr5* study. Leaf samples were taken 24 and 48 hpi for RNA extraction and gene expression analysis. This study identified 28 genes significantly induced during the development of resistance phenotypes across all eight *Yr* genes [13]. Among these transcripts, those for putative blue copper-binding protein, heat-stress transcription factor, pathogen-induced WIR1A protein, and ent-kaurene synthase transcripts were the most significant. Changed transcript levels were uniquely significant in each *Yr* gene line, indicating transcriptional events specific to particular *Yr* gene-mediated race-specific resistances. The results confirm the activity of known *R*-gene-mediated pathway race-specific resistance, including an oxidative burst that likely contributes to a hypersensitive response, as well as pathogenesis-related protein gene expression and activation of the phenylpropanoid pathway. 

## 4 COMPARISON OF TRANSCRIPTS IDENTIFIED FOR DIFFERENT GENES CONFERRING RACE-SPECIFIC ALL-STAGE RESISTANCE AND RACE NON-SPECIFIC HTAP RESISTANCE IN ADULT PLANTS UNDER HIGH TEMPERATURES

Similar to the meta-analysis of transcripts for race-specific resistance mediated by various genes, we also conducted a study to identify common transcripts associated with race non-specific HTAP resistance mediated by different genes in comparison with all-stage resistance. Isogenic lines having *Yr18*,* Yr29*,* Yr36 *and *Yr39* were selected as they were identified as single genes involved in race non-specific HTAP resistance. The isolines with the genes (*Yr1*,* Yr5*,* Yr7*,* Yr8*,* Yr9*,* Yr10*,* Yr15 *and *Yr17*) studied at the seedling stage [13] were also included. The custom microarray, experimental design, procedure, data collection and analyses were all as described for the race-specific resistance study [13], except that inoculated plants were grown in the high-temperature profile as described for the *Yr39* HTAP resistance study [10]. Boot stage adult plants of NILs *Yr1*,* Yr5*,* Yr7*,* Yr8*,* Yr9*,* Yr10*,* Yr15 *and *Yr17* were inoculated separately with appropriate avirulent races (PST-21 for *Yr8*, *Yr9*, *Yr10* and *Yr17*; PST-45 for *Yr1* and *Yr7*; and PST-78 for *Yr5* and *Yr15*) and virulent races (PST-17 for *Yr1*; PST-43 for *Yr10*; PST-45 for *Yr17*; PST-78 for *Yr7*, *Yr8* and *Yr9*; an Australian isolate for *Yr5*; and no isolate virulent for *Yr15*); and those of the single gene lines for *Yr18*,* Yr29*,* Yr36 *and *Yr39*, together with AvS, were inoculated with PST-78 that is virulent on seedlings, but not adult-plants, with these genes.

Stripe rust infection type data observed 20 days after inoculation were as expected for each compatible or incompatible *Yr* gene-*Pst* race combination, except the *Yr8*, *Yr10* and *Yr17* single gene lines in the presumed compatible interactions. Adult plants of these three lines exhibited resistance in the test with virulent races. The phenotypes of *Yr8* and *Yr17* confirmed the presence of HTAP resistance (Chen and associates, unpublished data), while that of the *Yr10* line was surprising. Because these lines have both all-stage and HTAP resistance, they were not included in the analyses of comparing transcripts of HTAP resistance with all-stage resistance.

For the race-specific adult-plant resistance gene lines, two probes [ribosomal protein L2 (Ta.28514.1) and mitogen-activated protein kinase (Ta.236.1)] were down-regulated when compared to their mock-inoculated checks and no probes were down regulated across the all-stage resistance gene lines compared to their compatible race inoculations. Four probes representing two transcripts [hydroxyproline rich glycoprotein (Ta.6952.1) and NB-ARC domain containing protein (TaAffx.103209.1)] were up-regulated when compared to their mock-inoculated checks and five probes representing 4 transcripts [hydroxyproline rich glycoprotein (Ta.6952.1), UDP-glucose dehydrogenase (Ta.2657.1), pathogen-induced WIR1A homolog (Ta.3133.1) and no homology (Ta.3247.1)] were up-regulated when compared to their compatible interactions. For the HTAP resistance gene lines, two probes representing one transcript [no homology (Ta.22462.1)] were down-regulated and four probes [UDP-glucose dehydrogenase (Ta.2657.1), pathogen-induced WIR 1A homolog (Ta.3133.1), no homology (Ta.3247.1) and gibberellin oxidase (Ta.24934.3)] were up-regulated when compared to AvS. When the HTAP resistance gene lines were compared with the all-stage resistance gene lines, nine probes representing six transcripts [no homology (Ta.22 462.1), hydroxyproline-rich glycoprotein (Ta.6952.1), NB-ARC domain containing protein (TaAffx.103209.1), no homology (TaAffx.27177.1), protein kinase (TaAffx.27775.1), no homology (Ta.22462.1)] were down regulated and two probes representing one transcript [nonclathrin coat protein (Ta.7616.1)] were up-regulated. 

Transcript values with significant changes (2-fold or higher, *P* <0.10) in the adult-plant tests under the high-temperature profile (10-30°C) are shown in bold in (Table **1**). Seven transcripts were significant for *Yr1*, 10 for *Yr5*, 4 for *Yr7*, 31 for *Yr8*, 6 for *Yr9*, 13 for *Yr10*, 6 for *Yr15*, 5 for *Yr17*, 40 for *Yr18*, 4 for *Yr29*, 99 for *Yr39* and none for *Yr36*. Up-regulated transcripts shared by two or more *Yr* genes also can be found from this table. For comparison, significant transcript values detected in previously published studies of seedling tests at the low-temperature (4-20°C) profile for all-stage resistance genes [8, 13] are also given in (Table **1**). In general, transcripts detected in the seedling low-temperature tests were also significant in the adult-plant high-temperature tests for race specific all-stage resistance.

**Fig. (2):**
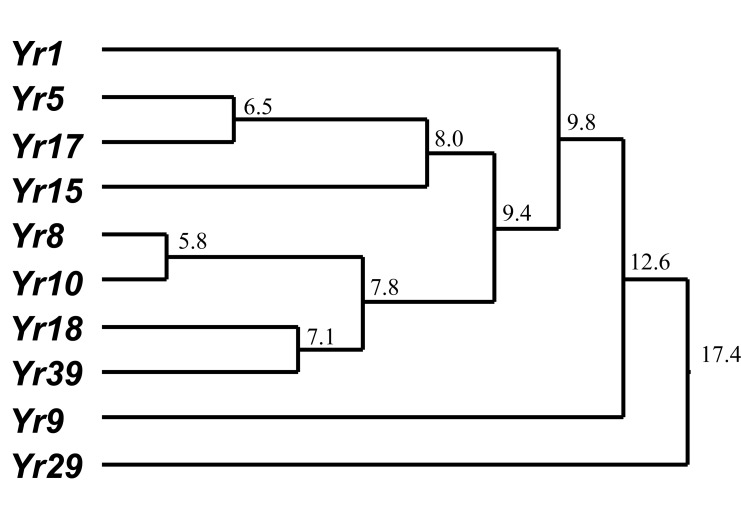
Hierarchical clustering (Euclidean metrics, complete linkage, distance indicated at each branch) of shared and unique data from (Table **1**).

Based on common and unique transcripts identified in the *Yr* gene-mediated resistances, a dendrogram was constructed to show their relationships (Fig. **2**). *Yr5* was more closely related to *Yr17*; *Yr8* was more closely related to *Yr10*; and *Yr18* was more closely related to *Yr39* than to other genes. *Yr29* was more distantly related to all of the other genes. *Yr36* was not included in the dendrograms as none of the transcripts for other genes was significantly changed in expression levels; this could indicate that it utilizes signaling and defense pathways that are different from those identified for the other genes. Although this hypothesis needs to be tested, the results may be in agreement with the previous finding that *Yr36* is a very old gene that is not present in common wheat cultivars [7].

After various comparisons, five genes were clearly identified to be involved in race-specific all-stage resistance controlled by *Yr1*, *Yr5*, *Yr7*, *Yr9* and *Yr15* and only one gene was commonly expressed in HTAP resistances mediated by different genes (*Yr18*, *Yr29*, *Yr36* and *Yr39*) (Table **2**). The annotation of the five transcripts specific to all-stage resistance provided additional evidence for classic *R*-gene mediated pathways being involved in race-specific resistance. The five genes commonly involved in all-stage resistance included a hydroxyproline-rich glycoprotein, a NB-ARC domain containing protein, a protein kinase and two function-unknown genes. In addition to the separate analysis at the seedling stage [13], we found another NB-ARC protein and a protein kinase for defense signaling. Also, the hydroxyproline-rich glycoprotein is involved in cell wall strengthening like the WIR1A protein [19]. Among the five genes, three were first identified in *Yr5*-mediated all-stage resistance and two were first identified in the *Yr39*-mediated HTAP resistance. The transcript with significant changes across all HTAP resistances is a nonclathrin coat protein. The function of nonclathrin coat protein-related resistance is not clear, but such proteins have been reported to bind to the cytoplasmic dilysine motif of membrane proteins of the early secretory pathway [20]. The nonclathrin coat protein identified in this study may be involved in transporting antifungal substances across the cell membrane to directly contact *Pst* haustoria or hyphae. The lack of many shared transcripts in HTAP resistance compared to all-stage resistance leads to a hypothesis that diverse genes and biochemical pathways are used by HTAP resistance controlled by different genes. Together with diverse genes and pathways identified for *Yr39*-mediated HTAP resistance [10], we conclude that highly diverse genes and biochemical pathways are the molecular basis for the race non-specificity and durability of HTAP resistance.

## 5 PERSPECTIVES AND CONCLUSIONS

The transcriptomics studies conducted so far have identified genes involved in *Yr5*-mediated all-stage resistance and *Yr39*-mediated HTAP resistance; and common genes involved in all-stage resistance and HTAP resistance mediated by different *Yr* genes. The results have provided some insights for understanding the molecular mechanisms of race-specific resistance compared to race non-specific resistance. In particular, the studies have linked a large number of genes with diverse functions to race non-specificity and durability of HTAP resistance. The data of these studies lead to several hypotheses to be tested and more studies to be conducted for a better understanding of various types of resistance and how to utilize the basic information to achieve more sustainable and better control of stripe rust.

We are currently conducting studies to test a hypothesis that ABC transporter proteins are involved in nonrace specific HTAP resistance. We are in the process of obtaining the full-length sequence for the identified ABC transporter gene, and so far have 5,754 bp of the genomic sequence from the *Yr39* donor, Alpowa, using a PCR based genome walking technique (Clontech GenomeWalker^TM^ Universal Kit, 638 904). Thus far, the ABC transporter-like wheat gene has more than 75% identity in genomic sequence to a rice gene, Os01g42410, with the greatest differences occurring in intron regions. Sequence conservation of the ABC transporter gene in Alpowa (*Yr39*) also appears to be high across different wheat cultivars after alignment of the newly acquired sequence with Chinese Spring genomic sequences (cerealsdb.uk.net) failed to detect significant differences, supporting the high up-regulation of the gene in the *Yr18* line presented above, as Chinese Spring has *Yr18* [6, 21]. Comparison of the ABC transporter gene in Alpowa with *Yr18*/*Lr34* [6, 21] shows that they have low nucleotide sequence similarity (39%), confirming our initial hypothesis that they are different genes. Future goals include obtaining the full-genomic sequence of the gene in addition to the 5’ untranslated region from Alpowa and other wheat cultivars to identify functional domains and single nucleotide polymorphisms (SNPs) potentially influencing HTAP resistance in Alpowa and other wheat cultivars.

The identification of the nine *R*-protein genes involved in *Yr39*-mediated HTAP resistance was initially a surprise to us, as R proteins are largely believed to be involved in recognition of pathogen effectors, leading to race-specific resistance. However, the high number of such types of genes leads us to believe that these genes collectively contribute to race non-specificity and therefore durability. Our hypothesis is that when up-regulated by the *Yr39* master gene, these genes serve as secondary master genes regulating other defense or functionally related genes to operate the entire defense machinery against *Pst* infection and growth in the plant tissue. These R proteins may recognize different effectors, which may make it difficult for the fungus to change to non-recognition. In regard to the ABC transporter gene, we are currently obtaining full-length sequences of these *R* genes to characterize them among wheat genotypes and to determine their functions for the HTAP resistance phenotype and their roles of being regulated or regulating in the total network of defense pathways.

With a primary goal of identifying transcripts commonly involved in all-stage resistance or HTAP resistance controlled by different genes, the custom microarray was constructed using genes identified in the *Yr5* and *Yr39* studies to represent those involved in either type of resistance. However, such a cost-saving approach did not allow us to identify transcripts uniquely involved in resistance mediated by individual *Yr* genes. Therefore, we still do not have the majority of the genes identified for all *Yr* genes studied, except for *Yr5* and *Yr39*. Using the Wheat Affymetrix GeneChip, which continues to have new genes added, is still a useful high-throughput technique to identify possible genes involved in resistance to stripe rust and other diseases in wheat. Alternately, transcriptome sequencing [22, 23], which can be used to determine numbers of transcripts for genes, should be useful to study genes involved in different types of resistance. 

HTAP resistance has two components: temperature sensitivity and developmental stage dependence. These two components are not equally required for resistance. Among cultivars with a broad-sense HTAP resistance, which can be determined by a virulent race in a seedling test under low temperatures and in a field or greenhouse test with the same race under high temperatures, resistance in some cultivars is more temperature sensitive whereas others are more plant-stage dependent. In our studies, *Yr39* is more typical of HTAP, where the maximum expression of resistance is in flag leaves and under high-temperatures. In contrast, both *Yr18* and *Yr36* can express resistance even in the seedling stage when under high temperatures [6, 7]. Thus it is likely that some transcripts involved in HTAP resistance respond more to temperatures, some more to growth stage, and others more or less neutral. In our studies to date this issue has not been addressed. Identification of genes responding to different environmental and growth stage conditions may allow choice of durable resistance genes that are more suited to specific regions in order to more effectively diversify resistance. 

In comparison of a pair of two resistance genes which regulate different transcripts with another pair of two resistance genes which regulate commonly shared transcripts, the former pair of resistance genes when in combination may lead to more durable resistance than the latter pair of genes as the combined resistance conferred by the former pair is based on more diverse defense pathways. Furthermore, the correct combinations of genes may provide higher levels of resistance. In this way, transcriptomics studies of resistance genes will not only provide an understanding of the basic mechanisms of resistance, but will allow for immediate application in selecting resistance genes for precision breeding. The results may revealed which genes are more likely to be durable and which are not. It will also add to the biotechnological tool box for identification of the same or different genes. As the technology is advancing, transcriptomics testing should become less expensive and higher throughput. It will become more feasible to use transcriptomics approaches for identifying genes and developing markers for different types of resistance. 

## Figures and Tables

**Table 1.** Shared and Unique Significantly (Changes > 2 fold; *P* < 0.10) Induced Transcripts for Stripe Rust Resistance Genes Conferring Race-Specific All-Stage Resistance Identified in Seedling Tests Under a Low Temperature Profile (4-20°C) and in Adult-Plant Tests Under a High-Temperature Profile (10-30°C) (Bold Script) Or Race Non-Specific High-Temperature Adult-Plant (HTAP) Resistance (Bold)

		Magnitude (fold) of Induced Expression of Resistance Gene*b*										
		Race-Specific*c*								Race Non-specific*d*		
Putative Function*a*	Probe ID	*Yr1*	*Yr5*	*Yr7*	*Yr8^e^*	*Yr9*	*Yr10*	*Yr15*	*Yr17*	*Yr18*	*Yr29*	*Yr39*
* Cell Death*												
Senescence-associated protein	Ta.14231.2.S1_x_at											**3.5**
* Defense*												
Alternative oxidase	Ta.10549.2.A1_at											**5.4**
Alternative oxidase	Ta.28112.1	**3.4**										
Alternative oxidase	Ta.28112.1.S1_at											**3.8**
Beta-1,3-glucanase	Ta.223.1							2.7	2.5			
Beta-1,3-glucanase	Ta.1174.1.S1_x_at		6.9									**7.0**
Beta-1,3-glucanase	Ta.21297.1.S1_at		4.8									
Beta-1,3-glucanase	Ta.21354.1.A1_at		2.1									**3.6**
Beta-1,3-glucanase	Ta.21354.1.A1_x_at		2.4									**3.6**
Beta-1,3-glucanase	Ta.22427.1	** 10.2**								**2.1**		
Beta-1,3-glucanase	Ta.22427.1.A1_x_at											**5.5**
Beta-1,3-glucanase	Ta.22562.1				**2.8**							
Beta-1,3-glucanase	Ta.26048.1.S1_x_at		3.0									
Beta-1,3-glucanase	TaAffx.119315.2.S1_at		2.6									
Beta-1,3-glucanase	TaAffx.119315.2.S1_x_at		2.3									
Cf2/Cf5 disease resistance protein homolog	Ta.25518.1.S1_at											**2.4**
Chitinase	Ta.30501.1.S1_at		9.5									
Cold-acclimation induced protein	Ta.351.2.S1_x_at											**2.8**
Dirigent-like protein	Ta.22687.1				**9.2**							
ERD1 protein - water-stress induced	Ta.4014.2.S1_at											**2.7**
Germin-like protein	TaAffx.15880.1.S1_at											**2.9**
Hydroxyanthranilate hydroxycinnamoyl transferase	Ta.14063.1.S1_at											** 2.5**
Leucine-rich repeat family protein	Ta.4479.2.S1_x_at											** 2.1**
Leucine-rich repeat transmembrane protein kinase	Ta.8590.1.S1_s_at											** 2.4**
MAC/perforin protein	Ta.12913.1									**2.7**	**2.3**	
Mitogen-activated protein kinase	Ta.236.1.S1_at											**2.4**
Mla6 barley powdery mildew resistance protein	Ta.14550.1				**2.9**							
NADPH-dependent HC-toxin reductase Hm1	Ta.12946.1.S1_at											**3.5**
NB-ARC domain containing protein	TaAffx.4601.2									**2.5**		
NB-ARC domain containing protein	TaAffx.103209.1.S1_at											**2.6**
NBS-LRR disease resistance protein	Ta.25549.1.S1_at											**2.7**
Pathogen induced WIR1A protein	Ta.13.1	**2.6**/2.3	**2.1**/2.0		**4.5**/4.1		**2.4**/2.1	**2.3**/2.2		**3.7**		
Pathogen induced WIR1B protein	Ta.97.1											**2.3**
Pathogen induced WIR1A protein	Ta.97.2.S1_x_at											**4.4**
Pathogen induced WIR1A protein	Ta.22732.1.S1_s_at											**4.8**
Pathogen-induced protein WIR1A homolog	Ta.22732.1.S1_x_at		3.6									**3.6**
Pathogen-induced protein WIR1A homolog	Ta.3133.1							**3.8**				**3.7**
Pathogen-induced protein WIR1A homolog	Ta.3133.1.S1_x_at		9.6									**5.0**
Pathogen-induced secretory protein	Ta.231.1				** 2.8**	** 2.4**	** 3.0**					
Peroxidase	Ta.82.1				** 3.4**		** 2.5**					
Peroxidase	Ta.18497.1					** 4.8**	** 9.9**			** 3.5**		** 3.0**
Peroxidase	Ta.18497.1.S1_at		5.6									
Peroxidase	Ta.21307.1.S1_x_at											** 6.5**
Peroxidase	Ta.22564.1						** 2.9**					
Peroxidase	Ta.24106.1.S1_x_at		3.1									
Phenylalanine ammonia-lyase	Ta.7022.1	** 4.6**/3.3										
Phenylalanine ammonia-lyase	Ta.7022.1.S1_at		2.5									**3.9**
Phenylalanine ammonia-lyase	Ta.7022.3	**11.5**/10.0	**10.3**/6.5	**10.3**/6.4								
Phenylalanine ammonia-lyase	TaAffx.131379.1		**2.9**									
Phenylalanine ammonia-lyase	TaAffx.131379.1.A1_at		5.4									
Phenylalanine ammonia-lyase	TaAffx.92008.1				**3.2**							
Phenylalanine ammonia-lyase	TaAffx.92008.1.A1_s_at		4.2									**3.3**
Phenylalanine ammonia-lyase	Ta.20429.1.S1_at											**4.6**
Phenylalanine ammonia-lyase	Ta.28046.1.A1_at											**3.8**
Phenylalanine ammonia-lyase	Ta.7022.1.S1_s_at											**8.6**
Phenylalanine ammonia-lyase	Ta.7022.1.S1_x_at											**3.8**
Pleiotropic drug resistance/ABC transporter	Ta.6990.1.S1_at											**2.4**
Pleiotropic drug resistance protein/ABC transporter	Ta.21281.1	**5.2**										**3.7**
PR protein 1	Ta.13013.2									**3.3**		
PR protein 10	Ta.22619.1	**3.2**/2.7			**11.5**/11.2		**3.5**/3.5					
PR protein 10	Ta.22619.1.S1_at		5.3									
PR protein 10	Ta.22619.1.S1_x_at		7.0									
Proline-rich protein	Ta.16599.1.S1_at		3.8									
Protein kinase	Ta.10236.1.A1_at											** 3.6**
Protein kinase	Ta.12007.2.S1_at											** 2.8**
Protein kinase - similar to barley stem rust R protein Rpg1	Ta.10236.2									** 3.8**		
Protein kinase - similar to barley Rpg1	Ta.10236.2.S1_a_at											** 4.7**
Protein kinase - similar to barley Rpg1	Ta.10326.1.S1_at											** 3.7**
Protein kinase - similar to barley Rpg1	Ta.10236.2.S1_x_at											**3.3**
Putative disease resistance protein	Ta.14786.1				**7.0**/6.0		**4.7**/4.3					
Putative disease resistance protein	Ta.14786.1.S1_at											**8.3**
Putative disease resistance protein	Ta.22482.1	**7.4**/4.7			**6.8**/6.6							
Putative disease resistance protein	Ta.22482.1.S1_s_at											**3.0**
Putative latex protein allergen	Ta.9588.2.S1_a_at											**4.8**
Putative stripe rust resistance protein Yr10	TaAffx.43336.1				**2.8**							
Putative stripe rust resistance protein Yr10	TaAffx.43336.1.S1_at											**2.4**
Receptor-like protein kinase	Ta.7017.1.S1_at											**2.8**
Receptor-like protein kinase	Ta.11135.1									**2.6**		
Receptor-like protein kinase	Ta.11135.1.S1_at											**2.5**
Receptor-like protein kinase	TaAffx.111955.1									**2.1**		
Receptor-like protein kinase	TaAffx.111955.1.S1_at											**3.2**
Reticuline oxidase	Ta.27350.1										**2.5**	
Serine/threonine protein kinase	Ta.728.1									**2.6**		
Serine/threonine protein kinase	Ta.7718.2.S1_a_at											**2.2**
Strictosidine synthase	TaAffx.56754.1.S1_at											**2.2**
Thaumatin-like protein	Ta.27762.1.S1_x_at		4.5									
UDP-glycosyltransferase	Ta.30731.1									**3.5**		
UDP-glucosyl transferase	Ta.8495.1.A1_at											** 8.3**
UDP-glucosyl transferase	TaAffx.23237.1.S1_at											** 3.3**
* Energy*												
Blue copper-binding protein	Ta.9336.1	**4.9**/4.6	**6.4**/5.1		**10.9**/7.1	3.1	**4.6**/4.1	**5.0**	**5.8**/4.0			
Blue copper-binding protein	Ta.18203.1		2.4					2.5	2.4			
Blue copper-binding protein	Ta.18203.1.S1_at											**3.3**
Blue copper-binding protein	Ta.5654.1.S1_at											**3.2**
Blue copper-binding protein	Ta.9336.1.S1_x_at		3.0									
Blue copper-binding protein	TaAffx.55612.1.S1_at											
Cytochrome P450	Ta.8262.1.S1_at		3.3									
Cytochrome P450	Ta.8447.1	**51.3**/16.8										
Cytochrome P450	Ta.8447.2	**3.4**					**4.1**					
Cytochrome P450	Ta.8447.1.S1_a_at		5.8									
Cytochrome P450	Ta.8447.1.S1_x_at		8.8									
Cytochrome P450	Ta.29826.1.S1_at		2.7									
Cytochrome P450	TaAffx.109794.1.S1_s_at		7.3									
* Growth*												
Ent-kaurene synthase	Ta.8418.1		**10.4**/6.9		**34.4**/23.8	**8.8**/6.8	**8.7**/8.1	**4.1**/3.5		**2.7**		**5.6**
Ent-kaurene synthase	Ta.8418.1.S1_at		3.0									
Gibberellin oxidase	Ta.24934.3		**5.6**		**3.0**							
Gibberellin oxidase	Ta.24934.3.S1_at		2.9									
* Metabolism*												
Acid phosphatase	Ta.21271.1									**2.7**		
Aspartyl protease	Ta.15123.1.A1_at											**2.1**
Bifunctional coenzyme A synthase	TaAffx.63502.1									**2.4**		
Beta-fructofuranosidase	TaAffx.82312.1.S1_s_at		2.2									
Glucosyl hydrolase	TaAffx.9022.1.S1_at											**4.7**
Prephenate dehydratase	Ta.9122.1.S1_x_at											**2.3**
Protein phosphatase	TaAffx.16090.1									**4.1**		
Protein phosphatase	TaAffx.16090.1.S1_at											**3.2**
Shikimate kinase	Ta.8618.1.S1_at											**3.0**
SIS domain protein	Ta.4815.1.S1_at		2.4									
UDP-glucose dehydrogenase	Ta.2657.1.S1_x_at		3.1									
* Signal transduction*												
Ankyrin-like protein	TaAffx.12271.1.S1_at											**2.7**
Calmodulin-binding heat shock protein	Ta.10168.1		**3.9**		**2.1**/4.4							
Calmodulin-binding heat shock protein	Ta.10168.1.S1_at											**2.1**
Calmodulin-binding protein	Ta.7711.1	**2.6**										
Calmodulin-binding protein	Ta.7711.1.A1_at											**4.0**
Calmodulin-like protein	TaAffx.128621.1.S1_at											**3.5**
GTP1/OBG family protein	Ta.16040.1									**2.6**	**2.1**	
LRR-containing extracellular glycoprotein	Ta.27314.1		**5.7**/4.6		**5.8**/4.8		**3.1**/2.9			**2.4**		
LRR-containing extracellular glycoprotein	Ta.27314.1.S1_at		3.5									
Protein kinase	TaAffx.27775.1									**2.4**		
Secretory protein kinase	TaAffx.52945.3		**3.3**		**2.7**			**2.6**				
Secretory protein kinase	TaAffx.52945.1.S1_at		3.7									
* Transcription*												
CR4-NOT transcription complex subunit 8 protein	TaAffx.33753.1.S1_at											**3.9**
Heat-stress transcription factor	TaAffx.120360.1	**5.0**/3.1		**2.3**/2.1	**4.8**/4.2	**3.5**/2.6	**2.3**/2.0	**2.1**/2.1		**2.1**		
Heat-stress transcription factor	TaAffx.120360.1.A1_at											** 2.6**
Putative WRKY5 protein	TaAffx.80313.1.S1_at											** 2.0**
Zinc finger DNA binding protein	TaAffx.28280.1									** 2.6**	** 2.1**	
Zinc finger POZ domain protein	Ta.19786.1				**2.5**							
* Transport*												
Ammonium transporter	Ta.27506.1				** 12.9**							**3.0**
Ammonium transporter	Ta.27506.1.S1_at											**4.0**
ATPase	TaAffx.54526.1									**2.3**		
ATPase	TaAffx.54526.1.S1_at											**5.0**
Glucose transporter	Ta.12517.1.S1_at		2.3									**3.7**
Histidine amino acid transporter	Ta.3869.1	**4.3**/3.6										
Histidine amino acid transporter	Ta.3869.1.S1_at											**4.8**
Histidine amino acid transporter	Ta.12339.1.S1_s_at											**2.1**
Histidine amino acid transporter	Ta.28479.1				3.4							
Histidine amino acid transporter	Ta.28479.1.S1_at											**3.2**
Integral membrane protein	Ta.15082.1.S1_at											**3.4**
Integral membrane protein	Ta.15082.1.S1_x_at											**3.1**
Integral membrane protein	Ta.29523.1.S1_at											**3.8**
Integral membrane protein	TaAffx.52897.1.S1_at											**3.9**
Nonclathrin coat protein	Ta.7616.1									**3.7**		
Phosphate transporter	Ta.10084.1.S1_at											**2.5**
Potassium transporter	Ta.9064.2.S1_s_at											**2.5**
Putative membrane protein	Ta.23392.1.S1_at											**4.8**
Putative peptide transporter	TaAffx.111465.1									**2.3**		
Sugar transporter	Ta.27329.1.S1_at		2.5									
UDP-galactose transporter	Ta.4921.1				**6.2**							
UDP-galactose transporter	Ta.4921.1.S1_at											**3.2**
* Unknown*												
Hypothetical protein	Ta.954.1.S1_s_at											**2.3**
Hypothetical protein	Ta.3088.1	**3.5**/3.1		**6.0**/3.5		**3.7**/3.1						
Hypothetical protein	Ta.13991.1.S1_x_at		3.3									**3.8**
Hypothetical protein	Ta.14129.1.S1_at											**3.9**
Hypothetical protein	Ta.14231.1.S1_x_at		2.4									
Hypothetical protein	Ta.22669.1.A1_at											**3.7**
Hypothetical protein	Ta.6155.2.S1_a_at											**2.2**
Hypothetical protein	Ta.13991.1.S1_x_at		**3.3**									**3.8**
Hypothetical protein	TaAffx.7032.1				**4.7**							
Hypothetical protein	TaAffx.7302.1.S1_at		2.7									
Hypothetical protein	TaAffx.107538.1								**3.6**	**2.3**		
Hypothetical protein	TaAffx.110081.1									**8.3**		
Hypothetical protein	TaAffx.107538.1.S1_x_at		2.8									
Hypothetical protein	TaAffx.110081.1.S1_at		2.7									
Hypothetical protein	TaAffx.110081.1.S1_x_at		2.9									
Hypothetical protein	TaAffx.110250.1.S1_x_at		2.8									
No homology	Ta.520.1.S1_at											**3.0**
No homology	Ta.424.1	**10.6**/8.6			**4.7**/4.3							
No homology	Ta.3247.1.S1_at		**2.1**									
No homology	Ta.4747.1			4.5	5.5			4.0				
No homology	Ta.5518.1.S1_at											**7.2**
No homology	Ta.8254.1.A1_at		3.5									
No homology	Ta.8582.1	**4.7**								**2.1**		
No homology	Ta.8582.1.S1_at		4.3									
No homology	Ta.8582.2.S1_a_at		4.5									
No homology	Ta.8582.2.S1_x_at		3.9									
No homology	Ta.11087.1				** 2.1**							
No homology	Ta.11087.2.S1_at		2.1									
No homology	Ta.11087.2.S1_x_at		2.7									
No homology	Ta.12795.1									** 5.9**		
No homology	Ta.15072.1									** 2.0**		
No homology	Ta.16472.1									**3.3**		
No homology	Ta.19411.1				**2.8**					**2.6**		
No homology	Ta.20149.1.S1_at											**4.0**
No homology	Ta.21236.1.S1_a_at		4.7									
No homology	Ta.21236.3.S1_x_at		5.5									
No homology	Ta.21314.1				**2.4**		**3.5**		**3.6**			
No homology	Ta.21314.1.S1_at		8.3									
No homology	Ta.21314.1.S1_x_at		8.1									
No homology	Ta.21531.1.S1_at											**2.7**
No homology	Ta.22223.1.S1_at		2.5									
No homology	Ta.22462.1				**4.0**							
No homology	Ta.22957.1.S1_at		5.9									
No homology	Ta.23271.1				**4.4**							
No homology	Ta.23271.2			**4.5**						**3.0**		
No homology	Ta.24564.1	**6.4**								**2.4**		
No homology	Ta.24564.1.S1_a_at											**2.4**
No homology	Ta.24564.1.S1_x_at											**2.6**
No homology	Ta.24564.3.S1_x_at											**2.3**
No homology	Ta.27882.1					**3.5**						
No homology	Ta.29516.1				**2.5**							
No homology	Ta.29984.1				**3.1**/3.0							
No homology	Ta.29984.1.S1_at											**5.1**
No homology	Ta.30753.1.A1_at											**2.5**
No homology	TaAffx.2056.1.A1_at											**4.1**
No homology	TaAffx.7236.1.S1_at											**4.4**
No homology	TaAffx.26815.1		3.2					3.3	3.3			
No homology	TaAffx.108203.1.S1_at											**3.7**
No homology	TaAffx.108939.1.S1_at		3.5									
No homology	TaAffx.109709.1.S1_at		2.6									
No homology	TaAffx.109765.1.S1_at		3.0									
No homology	TaAffx.110215.2.S1_at											** 5.9**
No homology	TaAffx.129395.1									** 2.4**		
No homology	TaAffx.23342.1								** 6.9**			
No homology	TaAffx.27177.1									** 2.1**		
No homology	TaAffx.27177.1.S1_at		2.7									
No homology	TaAffx.27427.1									**2.4**		
No homology	TaAffx.27427.1.S1_at											**2.0**
No homology	TaAffx.27956.1.S1_at											**2.8**
No homology	TaAffx.29213.1				**2.4**				**2.7**			
No homology	TaAffx.37517.1									**6.0**		
No homology	TaAffx.50112.1									**2.7**		
No homology	TaAffx.52926.1.S1_at		4.1									
No homology	TaAffx.55533.1.S1_at		2.3									**2.6**
No homology	TaAffx.56501.1.S1_at											**4.1**
No homology	TaAffx.59551.1									**2.5**		
No homology	TaAffx.64918.1									**4.5**		
No homology	TaAffx.82674.1		**4.3**/4.2									
No homology	TaAffx.82674.1.S1_at		2.1									
No homology	TaAffx.84007.1.S1_at											**3.1**

a Functional categories were based on the Munich Information Center for Protein Sequences classifications and putative function shows the best significant BLASTX database hit from HarvEST.

b Only expression values in fold change >2.0 (*P* < 0.10) of *Pst*-inoculated plants compared with mock-inoculated for each gene are presented. Values are combined for all time points.

c Inoculations were done at the two-leaf seedling stage and inoculated plants were grown at a low-temperature diurnal cycle (changing from 4°C at 2:00 a.m. to 20°C at 2:00 p.m.). Different races avirulent for these genes were used in inoculation.

d Inoculation were done at booting and inoculated plants were grown in a high-temperature diurnal cycle (changing from 10°C at 2:00 a.m. to 30°C at 2:00 p.m.). Race PST-78, which is virulent on seedlings of the HTAP resistance gene lines, was used in inoculation. The *Yr36* line was included in the study, but no significantly induced genes were identified.

e The *Yr8* near-isogenic line (AvSYr8NIL) also has a gene for race non-specific HTAP resistance linked to the race-specific all-stage resistance gene *Yr8*.

**Table 2.** Changes in Expression Levels of Transcripts Detected in Race-Specific All-Stage Resistances (*Yr1*, *Yr5*, *Yr7*, *Yr9* and *Yr15*) and in Race Non-Specific High-Temperature Adult-Plant (HTAP) Resistance (*Yr18*, *Yr29*, *Yr36* and *Yr39*)

Probe ID	Putative Function	Function Category	Origin	Mean log(2) Fold Change	*P* value
*Higher Expression in All-Stage Resistance*					
Ta.22462.1	No homology	Unknown	*Yr39* *Pst*-induced	2.13	0.000
Ta.6952.1	Hydroxyproline-rich glycoprotein	Defense - cell wall	*Yr5* incomplete isogenicity	3.43	0.000
TaAffx.103209.1	NB-ARC domain containing protein	Defense - R protein	*Yr39* HTAP-specific	1.15	0.006
TaAffx.27177.1	No homology	Unknown	*Yr5* HR-specific	1.28	0.000
TaAffx.27775.1	Protein kinase	Signal transduction	*Yr5* incomplete isogenicity	2.46	0.000
*Higher Expression in HTAP Resistance*					
Ta.7616.1	Nonclathrin coat protein	Transport	*Yr39* Pst-induced	1.11	0.000

## References

[1] Chen XM (2005). Epidemiology and control of stripe rust [*Puccinia
striiformis* f. sp. *tritici*] on wheat. Can. J. Plant Pathol.

[2] Stubbs RW, Roelfs AP, Bushnell WR (1985). Stripe rust. The Cereal Rusts, Vol. 2. Disease, Distribution, Epidemiology and Control.

[3] Line RF (2002). Stripe rust of wheat and barley in North America: A restrospective historical review. Annu. Rev. Phytopathol.

[4] Qayoum A, Line RF (1985). High-temperature, adult-plant resistance to stripe rust of wheat. Phytopathology.

[5] Chen XM (2013). High-temperature adult-plant resistance, a key for sustainable control of stripe rust. Amer. J. Plant Sci. Biotech.

[6] Krattinger SG, Lagudah ES, Spielmeyer W, Singh RP, Huerta-Espino J, McFadden H, Bossolini E, Selter LL, Keller B (2009). A putative ABC transporter confers durable resistance to multiple fungal pathogens in wheat. Science.

[7] Fu DL, Uauy C, Distelfeld A, Blechl A, Epstein L, Chen XM, Sela H, Fahima T, Dubcovsky J (2009). A kinase-START gene confers temperature-dependent resistance to wheat stripe rust. Science.

[8] Coram T, Wang MN, Chen XM (2008). Transcriptome analysis of the
wheat-*Puccinia striiformis* f. sp. *tritici* interaction. Mol. Plant Pathol.

[9] Coram TE, Settles ML, Wang MN, Chen XM (2008). Surveying expression level polymorphism and single-feature polymorphism in near-isogenic wheat lines differing for the *Yr5* stripe rust resistance locus. Theor. Appl. Genet.

[10] Coram TE, Settles ML, Chen XM (2008). Transcriptome analysis of high-temperature adult-plant resistance conditioned by *Yr39* during the wheat-*Puccinia striiformis* f. sp. *tritici* interaction. Mol. Plant Pathol.

[11] Coram TE, Brown-Guedira G, Chen XM (2008). Using transcriptomics to understand the wheat genome. CAB Reviews: Perspectives in Agriculture, Veterinary Science, Nutrition and Natural Resources.

[12] Coram TE, Settles ML, Chen XM (2009). Large-scale analysis of antisense transcription in wheat using the Affymetrix GeneChip wheat genome array. BMC Genom.

[13] Coram TE, Huang XL, Zhan GM, Settles ML, Chen XM (2010). Meta-analysis of transcripts associated with race-specific resistance to stripe rust in wheat demonstrates common induction of blue copper-binding protein, heat-stress transcription factor, pathogen-induced WIR1A protein, and ent-kaurene synthase transcripts. Func. Integ. Genom.

[14] Chen XM, Line RF (1992). Identification of stripe rust resistance genes in wheat cultivars used to differentiate North American races of *Puccinia striiformis*. Phytopathology.

[15] Yan GP, Chen XM, Line RF, Wellings CR (2003). Resistance gene analog polymorphism markers co-segregating with the *Yr5* gene for resistance to wheat stripe rust. Theor. Appl. Genet.

[16] Brueggeman R, Drader T, Kleinhofs A (2006). The barley serine/threonine kinase gene *Rpg1* providing resistance to stem rust belongs to a gene family with five other members encoding kinase domains. Theor. Appl. Genet.

[17] Dixon MS, Hatzixanthis K, Jones DA, Harrison K, Jones JDG (1998). The tomato *Cf-5* disease resistance gene and six homologs show pronounced allelic variation in leucine-rich repeat copy number. Plant Cell.

[18] Johal GS, Briggs SP (1992). Reductase activity encoded by the HM1 disease resistance gene in maize. Science.

[19] Bull J, Mauch F, Hertig C, Rebmann G, Dudler R (1992). Sequence and expression of a wheat gene that encodes a novel protein associated with pathogen defense. Mol. Plant-Microbe Interact.

[20] Harter C, Pavel J, Coccia F, Draken E, Wegehingel S, Tschochner H, Wieland F (1996). Nonclathrin coat protein gamma, a subunit of coatomer, binds to the cytoplasmic dilysine motif of membrane proteins of the early secretory pathway. Proc. Natl. Acad. Sci. USA.

[21] Krattinger SG, Lagudah ES, Wicker T, Risk JM, Ashton AR, Selter LL, Matsumoto T, Keller B (2011). *Lr34* multi-pathogen resistance ABC transporter: molecular analysis of homoeologous and orthologous genes in hexaploid wheat and other grass species. Plant J.

[22] Croucher NJ, Fookes MC, Perkins TT, Turner DJ, Marguerat SB, Keane T, Quail MA, He M, Assefa S, Bähler J, Kingsley RA, Parkhill J, Bentley SD, Dougan G, Thomson NR (2009). A simple method for directional transcriptome sequencing using Illumina technology. Nucleic Acids Res.

[23] Tuch BB, Laborde RR, Xu X, Gu J, Chung C (2010). Tumor transcriptome sequencing reveals allelic expression imbalances associated with copy number alterations. PLoS ONE.

